# LINC01140 Targeting miR-452-5p/RGS2 Pathway to Attenuate Breast Cancer Tumorigenesis

**DOI:** 10.1155/2022/2434938

**Published:** 2022-10-17

**Authors:** Xiaoshi Li

**Affiliations:** Department of Thyroid and Breast Surgery, The First Affiliated Hospital of Chengdu Medical College, Chengdu, 610041 Sichuan, China

## Abstract

**Background:**

LINC01140 has been known to be involved in various cancers. However, its underlying molecular mechanism in breast cancer (BC) needs further exploration.

**Methods:**

The LINC01140, miR-452-5p, and RGS2 levels in BC cells and tissues were evaluated by means of RT-qPCR and western blotting. The variations in the biological functions of BC cells were analyzed through CCK-8, transwell, western blotting, and xenograft experiments to observe cell viability, migration, levels of apoptosis-related proteins (Bax and Bcl-2), and tumor growth. The correlations existing among LINC01140, miR-452-5p, and RGS2 were validated through luciferase reporter and RIP assays.

**Results:**

LINC01140 and RGS2 were remarkably downregulated in BC cells and tissues, whereas miR-452-5p was upregulated. LINC01140 overexpression diminished BC cell viability, migration, and tumor growth and facilitated apoptosis. MiR-452-5p upregulation enhanced cell viability and migration and suppressed apoptosis. Nevertheless, the additional upregulation of LINC01140 could reverse the promotive effects of miR-452-5p upregulation. Additionally, RGS2 overexpression inhibited the malignant phenotypes of BC cells, but miR-452-5p upregulation abolished this effect. In terms of mechanisms, LINC01140 acted as a miR-452-5p sponge. Moreover, RGS2 was determined to be miR-452-5p's downstream target gene in BC.

**Conclusion:**

LINC01140 functioned as an antitumor agent in BC by sponging miR-452-5p to release RGS2. This hints that LINC01140 is a promising therapeutic target for BC.

## 1. Introduction

Breast cancer (BC) is among the most prevalent cancers among women around the world [1, 2]. It accounts for 18% of all cancer cases in women with approximately 1 million new cases reported worldwide each year [1, 2]. Over the past few decades, though, breast cancer mortality has declined steadily, thanks to improved screening and treatment [3]. But because this type of cancer can spread to distant organs or lymph nodes, it is still considered the leading cause of death in women [4, 5]. Traditional therapies, including chemotherapy, endocrine therapy, and radiotherapy, do not cure BC most of the time but only improve clinical outcomes [6]. Hence, it is urgent to develop effective approaches for BC diagnosis and therapy.

Long noncoding RNAs (lncRNAs) are larger than 200 bp and have no protein-coding ability [7]. Previous studies have found that abnormal lncRNA expression often has a specific pattern in human tumor tissues [8]. Over the recent years, there has been accumulating evidence that lncRNA is new cancer mediator that can regulate gene expression through transcription, posttranscriptional, or epigenetic levels, in order to participate in almost all malignant behaviors of tumor cells [8, 9]. For example, the newly discovered LINC02273 is significantly elevated in metastatic BC lesions and is an independent prognostic factor in predicting patient survival [10]. LncRNA HOTAIR induces BC cell survival and optimizes drug resistance [11]. LINC01140 is a potential regulator of cancer drive or containment in different tumors. LINC01140 inhibits the progression of lung cancer [12] and sarcomas [13], whereas it promotes bladder cancer. Li et al. have analyzed the cBioPortal database and reported that LINC01140 expression is significantly reduced in the tumor samples from BC patients [14]. This predicted a worse recurrence-free survival rate of BC patients [14]. Nevertheless, the underlying mechanisms of LINC01140 need to be researched further.

microRNAs (miRNAs) are small RNA molecules that bind to targets of mRNA, acting as gene silencing and translation inhibitors [15]. Over the past few years, the function of miRNAs in biological processes and in the occurrence and development of various human diseases, including cancer, has been extensively studied [16, 17]. MiR-452-5p has been proven to be a regulatory factor of colorectal cancer, liver cancer, and lung cancer, specifically promoting the invasion, migration, and proliferation of cancer cells [18, 19]. Furthermore, the elevated miR-452-5p expression in squamous cell carcinoma has been reported to have a significant involvement in tumor lymph node metastasis [20]. More importantly, one analysis has indicated that miR-452-5p is aberrantly expressed in BC [21]. Hence, I speculate that miR-452-5p may play an important role in BC. I expect to further explore miR-452-5p's influence on BC development and its specific regulatory mechanism.

Regulator of G-protein signaling 2 (RGS2) is a member of the GTPase activating protein (GAP) family of Ga subunits. It has been originally identified as an inhibitor of G protein signal transduction, but recent studies have shown that it has cellular proliferation regulatory functions as well [22]. RGS2 is highly expressed in normal human cells but downregulated in cancer cells, including breast adenocarcinoma, wherein it plays a tumor suppressive function [23, 24]. Therefore, in-depth study of the effects of RGS2 may improve my knowledge of the pathogenesis of BC.

The potential mechanism of LINC01140 in BC needs to be analyzed in detail. I hypothesize that the LINC01140/miR-452-5p/RGS2 axis is a new signaling pathway related to the progression of BC. This may provide valuable theoretical basis for BC diagnosis and treatment.

## 2. Methods

### 2.1. Tissues Samples Collection

Thirty-eight [25] sets of tumoral and normal adjacent tissues were acquired from BC patients. All patients that received surgery in the First Affiliated Hospital of Chengdu Medical College provided a signed informed consent and did not undergo radiotherapy or chemotherapy prior their procedure. The cancer tissues were confirmed by at least two pathologists. The Ethics Committee of the First Affiliated Hospital of Chengdu Medical College approved this study.

### 2.2. Cell Culture

Three of BC cell lines (MDA-MB-231, MCF-7, and HCC1937) and MCF-10A were all obtained from ATCC (USA). DMEM (Gibco, USA) containing 1% P/S as well as 10% FBS (Gibco) was utilized for the culturing of cells. The cultures were maintained at 37°C in humidified atmosphere that contained 5% CO_2_. Cells were digested and subcultured when the confluence reached 80-90%. The third-generation cells at logarithmic growth phase were chosen for the succeeding studies.

### 2.3. RT-qPCR Assay

MiRNA was isolated using the NucleoSpin® miRNA kit (Macherey Nagel, France), and total RNA was extracted with the aid of the RNA isolation kit (Takara, Japan). ImProm-II reverse transcription system (Promega, USA) and PrimeScript™ RT master mix (Takara) were utilized for miRNA and RNA reverse transcription, respectively. Subsequently, LightCycler 480 System (Roche, Germany) with the miRcute miRNA qPCR detection kit (Tiangen) or TaqMan universal master mix (Applied Biosystems) was used for the miRNA and RNA qPCR assay in a CFX connect real-time PCR detection system (Bio-Rad, USA). The RNA and miRNA levels were computed by applying the 2^-*ΔΔ*Ct^ formula, and the results were normalized against GAPDH and U6, respectively [26].  Table 1 lists the primers used.

### 2.4. Subcellular Localization

Following the included protocol, the PARIS kit (Thermo Fisher Scientific, USA) was employed to isolate and collect the nucleus and cytoplasm of MDA-MB-231 and HCC1937 cells. The LINC01140 expressions in the nucleus and cytoplasm were assessed via RT-qPCR and then normalized against U6 and GAPDH, respectively.

### 2.5. Cell Transfection

LINC01140 and RGS2 overexpression plasmids (LINC01140-OE and RGS2-OE), as well as the empty vector, were provided by GeneChem (Shanghai, China). The plasmids were utilized for the overexpression of LINC01140 and RGS2, and the empty vector served as the control. SwitchGear Genomics (USA) offered the miR-452-5p mimic and its negative control, mimic-NC. Lipofectamine 2000 (Invitrogen, USA) was utilized in transfecting the MDA-MB-231 and HCC1937 cells with 100 nM mimic or 2 *μ*g/ml OE RNA. After even and careful shaking, the culture was incubated at 37°C for 48 h. Afterward, RT-qPCR assay was performed to evaluate the transfection efficiency.

### 2.6. CCK-8 Assay

CCK-8 kit (Sigma, USA) was utilized to observe the viabilities of the HCC1937 and MDA-MB-231 cells. Simply put, 5 × 10^4^ cells were plated on each of the 96-wells of a culture plate and then cultivated for 0, 24, 48, and 72 h. Afterward, CCK-8 (10 *μ*l) was added and incubated with the transfected cells at 37°C for 2 h. The wavelengths were determined using the FLx800 fluorescence microplate reader (BioTek, USA) with a 450-nm filter.

### 2.7. Transwell Migration Assay

Transwell inserts (Corning, USA) were utilized in performing this assay. Complete medium (500 *μ*l) was poured into the lower compartments of the inserts. Meanwhile, 1 × 10^5^ treated cells/ml were mixed with a medium that was serum-free and then pipetted into the upper chambers. After 24 h of culturing, the migrated cells were immobilized using 4% methanol for 15 min and then dyed with 0.25% crystal violet for 10 min. The cells that migrated were photographed and tallied with the help of a microscope (Olympus, Japan).

### 2.8. Western Blot

Proteins were isolated from the MDA-MB-231 and HCC1937 cell lines with the aid of a RIPA lysis buffer (Invitrogen). Their concentrations were then quantified with a BCA kit (Thermo Fisher). The protein samples were subjected to electrophoresis on a 10% SDS-PAGE gel before they were moved onto PVDF membranes. They then were blocked with 5% nonfat milk for 1 h at 25°C. Subsequently, the proteins were incubated overnight at 4°C with the following antibodies from Abcam: anti-Bax (1 : 1000, ab32503), anti-Bcl-2 (1 : 1000, ab32124), anti-RGS2 (1 : 1000, ab155762), and anti-GAPDH (1 : 2000, ab181603). Afterward, they were supplemented with the corresponding secondary antibody (1 : 2000, ab97051, Abcam) and maintained at room temperature for 2 hours. At last, the protein blots were visualized with the aid of an ECL Reagent (GE Healthcare, USA).

### 2.9. Xenograft Tumor Model

HCC1937 cells (1 × 10^6^) stably transfected with LINC01140-OE or empty vector were subcutaneously injected into ten BALB/c mice (5 weeks old, five mice/group) acquired from Hunan SJA Laboratory Animal (China). The tumors were measured every 4 days, and their volumes were calculated using the following formula: volume = (length) × (width)^2^/2. The mice were sacrificed 28 days after the inoculation of tumor cells, and the tumor xenografts were excised then weighed. The Animal Care and Use Committee of the First Affiliated Hospital of Chengdu Medical College has authorized this assay.

### 2.10. Luciferase Reporter Assay

Wild-type LINC01140 (LINC01140-WT) or RGS2 3'UTR (RGS2-WT) sequences with miR-452-5p binding sites were inserted into pGL3 reporter constructs (Promega). Mutant LINC01140 (LINC01140-MUT) or RGS2 (RGS2-MUT) were obtained with the QuikChange site-directed mutagenesis kit (Stratagene, USA). Lipofectamine 2000 was then utilized to deliver the reporter constructs, along with a mimic-NC or miR-452-5p mimic, into the HCC1937 and MDA-MB-231 cells. Forty-eight hours later, the luciferase activities of the different vectors were revealed by the dual-luciferase report analysis system (Promega, USA).

### 2.11. RIP Assay

This assay was conducted using the BersinBioTM RIP kit (BersinBio, China). Magnetic beads were pretreated with Ago2 and negative control IgG antibody. Afterward, they were conjugated with prepared HCC1937 and MDA-MB-231 cell lysate suspension for 4 h at 4°C. Protein A-Sepharose was then added to the product and then maintained at 4°C for 4 h. Finally, RT-qPCR was conducted to assess LINC01140 and miR-452-5p levels.

### 2.12. Statistical Analysis

The experimental data were expressed as the mean ± SD and were analyzed in GraphPad Prism 8.0 (GraphPad Software, USA). Correlation analyses between miR-452-5p and LINC01140 or RGS2 levels in cancer tissues were determined by Pearson's correlation coefficient. *P* < 0.05 indicated statistical significance. Student's *t*-test was used to test the variations between two groups, whereas one-way ANOVA was applied for multiple groups.

## 3. Results

### 3.1. LINC01140 Suppressed BC Cell Migration and Proliferation, Facilitated Apoptosis, and Inhibited *in* vivo Tumor Growth

**Figure 1:**
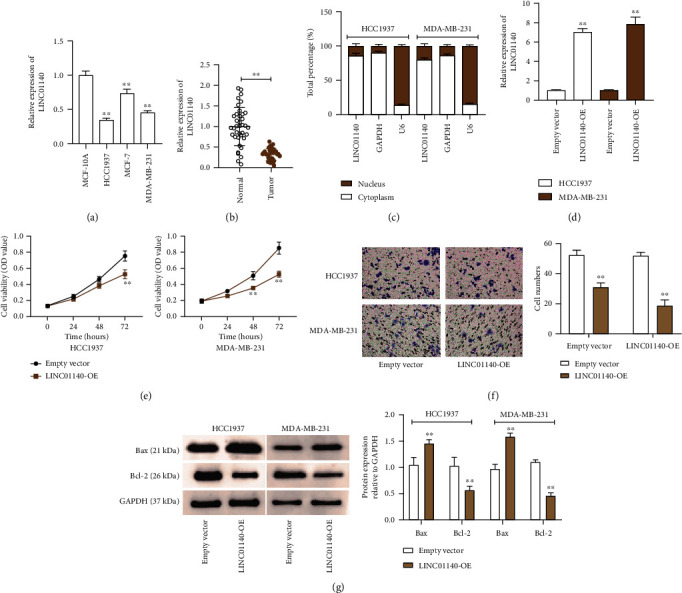
LINC01140 overexpression suppressed BC cell proliferation and migration and facilitated apoptosis *in vitro*. (a) RT-qPCR was utilized for measuring LINC01140 expression in normal MCF-10A cells and BC cells (HCC1937, MCF-7, and MDA-MB-231). ^∗∗^*P* < 0.001 vs MCF-10A. (b) RT-qPCR was utilized for measuring LINC01140 level in BC tissues and adjacent tissues. ^∗∗^*P* < 0.001. (c) Subcellular localization was utilized for measuring LINC01140 expression in cytoplasmic and nuclear of HCC1937 and MDA-MB-231 cells. (d) HCC1937 and MDA-MB-231 were transfected with LINC01140 overexpression vector (LINC01140-OE) and its corresponding negative control (empty vector). LINC01140 expression in these transfected cells was measured by RT-qPCR. ^∗∗^*P* < 0.001 vs empty vector. (e) Cell viability in HCC1937 and MDA-MB-231 cells delivered empty vector and LINC01140-OE, as uncovered utilizing CCK-8 assay. ^∗∗^*P* < 0.001 vs empty vector. (f) Migrated cells were counted utilizing transwell in HCC1937 and MDA-MB-231 cells delivered empty vector and LINC01140-OE. ^∗∗^*P* < 0.001 vs empty vector. (g) Bax and Bcl-2 protein levels was uncovered utilizing western blotting analysis in HCC1937 and MDA-MB-231 cells delivered empty vector and LINC01140-OE. ^∗∗^*P* < 0.001 vs empty vector.

As illustrated in Figure 1(a), the expressions of LINC01140 in MCF-7, MDA-MB-231, and HCC1937 cells are 25%, 55%, and 75% lower, respectively, than that of the MCF-10A cells. I also determined LINC01140 levels in clinical tissues, and the data manifested that the LINC01140 levels in BC specimens were approximately 80% lower than that of the normal tissues (Figure 1(b)). Subcellular localization experiment was conducted to identify the location of LINC01140 in BC cells. The outcome of the experiment revealed that LINC01140 predominantly existed more within the cytoplasm than in the nucleus. This suggests that LINC01140 may perform posttranscriptional and transcriptional regulatory functions in BC cells (Figure 1(c)). Based on these results, I clarified LINC01140's influence on the behavior of BC cells. LINC01140 overexpression vector was delivered into the HCC1937 and MDA-MB-231 cells. Thereafter, LINC01140 levels increased by more than 7-fold in the BC cell lines in contrast to that in the empty vector group (Figure 1(d)). CCK-8 analysis revealed that the overexpression of LINC01140 reduced cell viability by about 40% (Figure 1(e)). Moreover, the transwell experiment demonstrated that LINC01140 upregulation reduced the number of migrating cells by over 45% (Figure 1(f)). In addition, I also observed an increase in Bax protein levels and a decrease of Bcl-2 in the LINC01140-OE groups (Figure 1(g)). These abovementioned results manifest that LINC01140 overexpression effectively represses BC cell survival *in vitro*.

**Figure 2:**
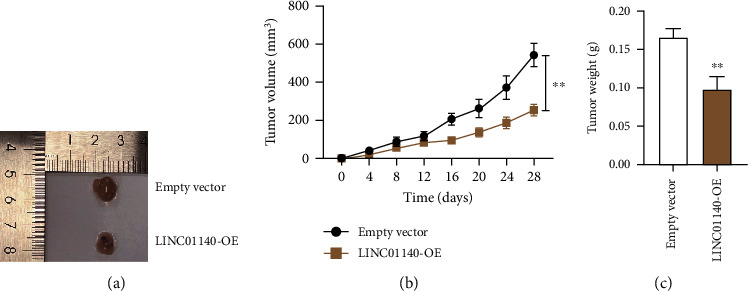
LINC01140 overexpression inhibited BC cells tumorigenicity *in vivo*. Nude mice were injected with HCC1937 cells stably transfected with empty vector and LINC01140-OE (five mice/group). (a) Representative tumor images of nude mice. (b) Tumor growth curve of nude mice from day 0 to day 28. (c) Tumor weight of nude mice. ^∗∗^*P* < 0.001 vs empty vector.

I further explored LINC01140's impact on the growth of BC cells *in vivo*. HCC1937 cells from the LINC01140-OE or empty vector groups were administered into the nude mice. In comparison with empty vector groups, the tumor sizes and volumes among the nude mice from the LINC01140-OE group were reduced (Figures 2(a) and 2(b)). Weighing the tumors further revealed that LINC01140 upregulation diminished the weight of the tumors (Figure 2(c)). These suggest that the ectopic expression of LINC01140 may inhibit the growth of BC cells *in vivo*.

### 3.2. LINC01140 Sponged miR-452-5p

**Figure 3:**
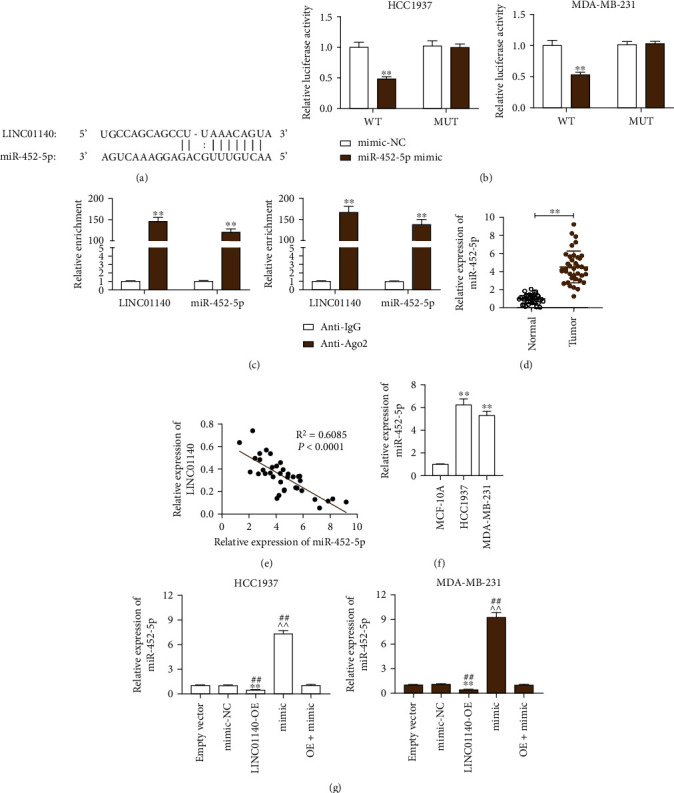
LINC01140 sponged miR-452-5p (a) StarBase was utilized for the prediction of miR-452-5p binding sites on LINC01140. (b) Luciferase reporter experiment was conducted to measure the luciferase activities among MDA-MB-2312 and HCC1937 cells that had a combined transfection of a miR-452-5p mimic or miR-NC plus either a LINC01140-WT or LINC01140-MUT reporter plasmid. ^∗∗^*P* < 0.001 vs mimic-NC. (c) MiR-452-5p and LINC01140 enrichment levels on anti-Ago2 bind magnetic beads, as determined via RIP assay. ^∗∗^*P* < 0.001 vs anti-IgG. (d) RT-qPCR was utilized for measuring miR-452-5p expression in BC as well as adjacent tissues. ^∗∗^*P* < 0.001. (e) The association between the expressions of miR-452-5p and LINC01140 in BC tissues was analyzed by Pearson analysis. (f) RT-qPCR was utilized for the determination of miR-452-5p expression in normal MCF-10A cells and BC cells (HCC1937 and MDA-MB-231). ^∗∗^*P* < 0.001 vs MCF-10A. (g) RT-qPCR was utilized for measuring miR-452-5p expression in empty vector, mimic-NC, LINC01140-OE (OE), mimic or OE+mimic transfected HCC1937, and MDA-MB-231 cells. ^∗∗^*P* < 0.001 vs empty vector; ^^*P* < 0.001 vs mimic-NC; ##*P* < 0.001 vs OE+mimic.

In virtue of starBase, the miRNAs binding to LINC01140 were predicted (Supplementary table 1). Due to the inhibitory effect of miR-452-5p in multiple cancers, I selected miR-452-5p to explore its regulatory mechanism in breast cancer. The binding site between LINC01140 and miR-452-5p sequences is shown in Figure 3(a). Next, I explored the binding relationship between LINC01140 and miR-452-5p. Results of the luciferase assay showed that the miR-452-5p ectopic expression only reduced LINC01140-WT luciferase activity, whereas that of LINC01140-MUT did not change (Figure 3(b)). Additionally, it was uncovered via RIP experiment that miR-452-5p and LINC01140 were enriched in compounds precipitated by anti-Ago2 antibodies (Figure 3(c)). This implies that miR-452-5p binds to LINC01140. Hence, I assumed that miR-452-5p was abnormally expressed in BC. It was shown via RT-qPCR that miR-452-5p levels in BC were approximately 4.6-fold of that of the normal samples (Figure 3(d)). Pearson correlation analysis then confirmed an inverse association between LINC01140 and miR-452-5p levels in BC samples (Figure 3(e)). Additionally, I also observed significantly elevated miR-452-5p levels among the BC cells lines compared to MCF-10A cells (Figure 3(f)). Moreover, I studied the regulatory effect of LINC01140 on miR-452-5p and learned that the expression of miR-452-5p was downregulated by more than 80% after LINC01140-OE transfection. Furthermore, the miR-452-5p upregulation effected by the miR-452-5p mimic, which was over 7.5-fold of the control, was also reversible by LINC01140-OE (Figure 3(g)). In a summary, miR-452-5p was LINC01140's target gene.

### 3.3. LINC01140/miR-452-5p Blocked the Malignant Behavior of BC Cells

**Figure 4:**
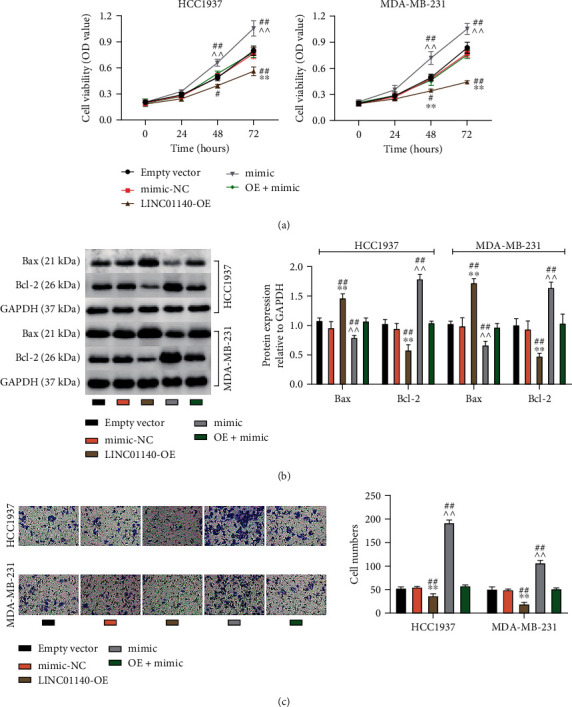
LINC01140 blocked the malignancy behavior of BC cells by targeting miR-452-5p. MDA-MB-231 and HCC1937 cells were transfected with an empty vector, mimic-NC, LINC01140-OE (OE), mimic, or OE+mimic. (a) Cell viability in these above transfected BC cells was uncovered utilizing CCK-8 assay. (b) Bax and Bcl-2 protein levels in these above transfected BC cells were uncovered utilizing western blotting analysis. (c) Migrated cells were counted utilizing transwell in these above transfected BC cells. ^∗∗^*P* < 0.001 vs empty vector; ^^*P*.<0.001 vs mimic-NC; #*P* < 0.01, ##*P* < 0.001 vs OE+mimic.

In a follow-up study, I used a salvage trial to verify the role of LINC01140/miR-452-5p in BC. First, as shown in Figure 4(a), CCK-8 reveals that miR-452-5p overexpression increased cell viability by about 1.4 times, but the extra LINC01140 upregulation offsets this effect on cell viability. In addition, miR-452-5p mimic inhibited Bax protein levels and upregulated Bcl-2 levels, but this was reversed by the enhanced apoptotic effect of LINC01140-OE (Figure 4(b)). Moreover, in transwell analysis, cell migration levels were found to be upregulated by at least 2-fold after miR-452-5p mimic transfection. However, LINC01140 overexpression restored this upregulation to normal levels (Figure 4(c)). In summary, I conclude that LINC01140 modulates miR-452-5p to achieve its anticancer function in BC cells.

### 3.4. RGS2 Was miR-452-5p's Downstream Target

**Figure 5:**
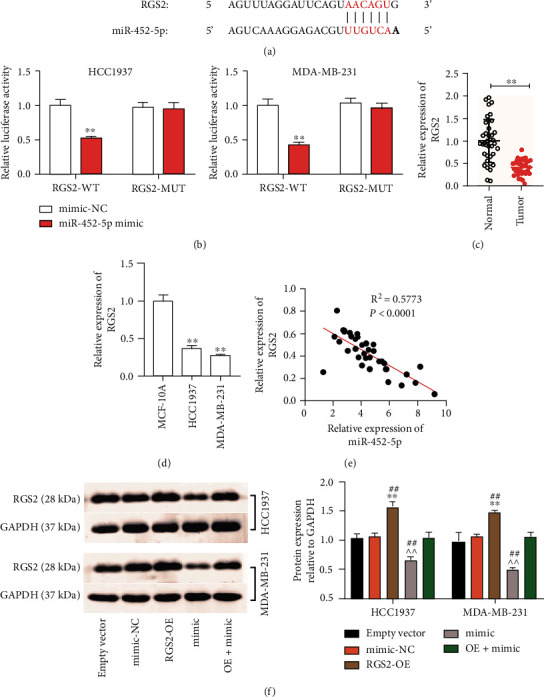
RGS2 was miR-452-5p's downstream target. (a) StarBase was utilized in the prediction of the miR-452-5p binding sites on RGS2. (b) Luciferase reporter experiment was conducted to assess the luciferase activities in the MDA-MB-2312 and HCC1937 cells that had a combined transfection of RGS2-WT or RGS2-MUT reporter plasmid plus either a miR-452-5p mimic or miR-NC. ^∗∗^*P* < 0.001 vs mimic-NC. (c) RT-qPCR was utilized for measuring RGS2 expression in BC as well as adjacent tissues. ^∗∗^*P* < 0.001. (d) RT-qPCR was utilized for the determination of RGS2 expression in normal MCF-10A cells and BC cells (MDA-MB-231 and HCC1937). ^∗∗^*P* < 0.001 vs MCF-10A. (e) The association between miR-452-5p and RGS2 levels in BC tissues was ascertained by Pearson's *r*. (f) Western blotting was utilized for measuring RGS2 protein expression in empty vector, mimic-NC, RGS2-OE (OE), mimic or OE+mimic transfected HCC1937, and MDA-MB-231 cells. ^∗∗^*P* < 0.001 vs empty vector. ^^*P* < 0.001 vs mimic-NC. ##*P* < 0.001 vs OE+mimic.

To explore the downstream of miR-452-5p, I utilized starBase to forecast its target genes. I discovered that RGS2 has a binding site for miR-452-5p (Figure 5(a)). Targeting analyses revealed that RGS2-WT group decreased the luciferase activity by 55% after the transfection of the miR-452-5p mimic. Meanwhile, no significant changes in luciferase activity were observed in the RGS2-MUT group (Figure 5(b)). Moreover, the expression of RGS2 mRNA in BC tissues was 45% of that in normal tissues (Figure 5(c)). I further found that HCC1937 and MDA-MB-231 cells manifested RGS2 levels that were 60% and 70% lower, respectively, than that in the MCF-10A cells (Figure 5(d)). Also, miR-452-5p levels were inversely correlated with RGS2 levels (Figure 5(e)). Western blotting showed that miR-452-5p mimic treatment downregulated RGS2 protein levels. Nevertheless, this change could be reversed by RGS2 overexpression (Figure 5(f)). These results suggest that miR-452-5p targets and negatively regulates RGS2.

### 3.5. MiR-452-5p Facilitated BC Cells Survival by Regulating RGS2

**Figure 6:**
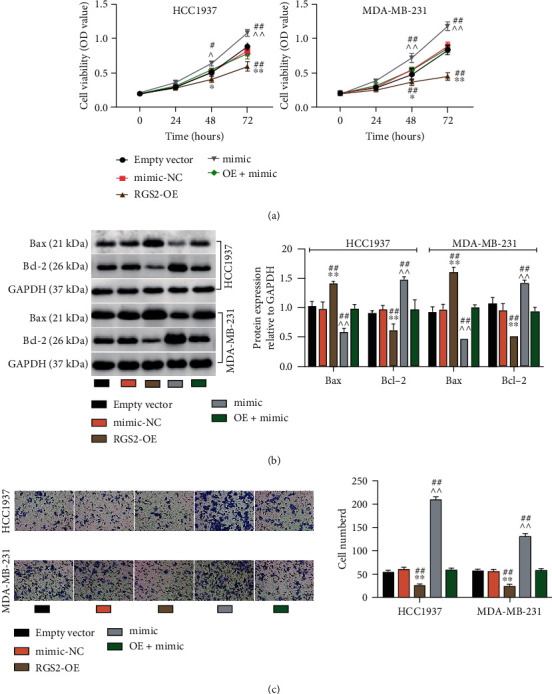
MiR-452-5p facilitated BC cells survival by downregulating RGS2. MDA-MB-231 and HCC1937 were transfected with an empty vector, mimic-NC, RGS2-OE (OE), mimic, or OE+mimic. (a) Cell viability in BC cells transfected as described above was uncovered utilizing CCK-8 assay. (b) Bax and Bcl-2 protein levels were uncovered utilizing western blotting analysis in BC cells transfected as described above. (c) Migrated cells were counted utilizing transwell in BC cells transfected as described above. ^∗^*P* < 0.05; ^∗∗^*P* < 0.001 vs empty vector; ^∗^*P* < 0.05, ^^*P* < 0.001 vs mimic-NC; #*P* < 0.05, ##*P* < 0.001 vs OE+mimic.

I scrutinized the miR-452-5p and RGS2's interaction in malignant BC phenotype. CCK-8 demonstrated that in contrast to the empty vector group, cell viability in the RGS2-OE group was lower, but it was also partially reversible through the introduction of the miR-452-5p mimic (Figure 6(a)). In addition, after RGS2 upregulation, Bax protein levels increased while that of Bcl-2 decreased. However, miR-452-5p mimic could considerably mitigate the proapoptosis effect engendered by RGS2 upregulation (Figure 6(b)). The transwell experiments showed that the cell migration level diminished after RGS2-OE transfection. Meanwhile, miR-452-5p mimic alleviated the repressive influence of RGS2 upregulation on cell migration (Figure 6(c)). These reveal that RGS2 restrains the aggressive behaviors of BC cells but is mediated by miR-452-5p.

## 4. Discussion

BC has killed about 630,000 people worldwide, according to Globocan data in 2018 [27]. Despite significant advances in cancer medicine over the past decade, my understanding of BC progression remains limited [28]. It would be helpful to explore the genetic characteristics of BC and its relationship with tumor progression to guide the clinical treatment and diagnosis of BC [29]. In my research, I observed that LINC01140 was remarkably downregulated in BC cells and tissues. Moreover, its overexpression inhibited BC cell viability, migration, and tumor growth, while promoting apoptosis. LINC01140 may be a tumor suppressor associated with BC. In terms of mechanisms, LINC01140 acts as miR-452-5p'sponge, releasing RGS2. These results may contribute theoretical bases for the clinical diagnosis and treatment of BC.

LINC01140 is one of the few new lncRNAs identified to be involved in tumor prognosis. Hu et al. [13] have discovered that the low levels of LINC01140 in metastatic sarcomas indicate disease-free survival, disease-specific survival, and poor overall survival. Meanwhile, its high expression may promote survival of sarcomas. Inconsistently, low LINC01140 inhibits the survival and metastasis of glioma cells. A study by Wu *et al*. [30] revealed that LINC01140 was significantly upregulated in muscle-invasive bladder cancer. Furthermore, its downregulation inhibited the invasive ability of cancer cells [30]. This may be caused by the tissue specificity of LINC01140, suggesting that LINC01140 may operate as a tumor suppressor or progenitor in different tumor types. Herein, my experimental results confirmed the previous report by Li et al. [14] that LINC01140 expression was significantly reduced in BC. Furthermore, in the present study, I found that the overexpression of LINC01140 inhibited the malignant behavior of BC cells *in vitro* and reduced tumor growth in nude mice xenografts. This reflects that LINC01140 may serve as a potential regulator gene for the suppression of BC.

A growing number of researchers are paying attention to the role of miRNAs in human diseases and cancer [31]. Some works have documented that miRNA dysregulation has a crucial part in the progression of BC [32]. It can be used as a diagnostic marker for BC and is related to the treatment resistance of BC [32]. For example, the miR-205 expressions among the different subtypes of BC, ranging from the less aggressive subtype to the more aggressive triple negative breast cancer (TNBC), affect metastatic ability, treatment response, and patient survival [33]. MiR-9 levels may be used as a potential noninvasive tumor marker for BC [34]. Li et al. [35] reported that the results of the next-generation sequencing showed the overexpression of miR-452 BC tissues. Meanwhile, their RT-qPCR results revealed the low levels of miR-452 levels in TNBC. The researchers also reported the inhibitory influence of miR-452 antagonists on TNBC xenografts. Herein, my findings show high expressions of miR-452-5p in BC, which is similar to the next-generation sequencing results of Li *et al*. [35], but contrary to the results of RT-qPCR detection. Nevertheless, it is speculated that the difference in the aggressiveness of the cancer tissue may be responsible for this. MiR-452-5p is an oncogenic factor in colorectal cancer, liver cancer, and lung cancer. It promotes cell proliferation, cell cycle transformation, and chemotherapy resistance, but inhibits apoptosis [18–20]. In contrast, however, miR-452-5p inhibits the development of prostate and cervical cancers [36, 37]. These may be due to the heterogeneity of the cancers. The outcomes of this study are consistent with those in colorectal cancer, liver cancer, and lung cancer. I have learned that the miR-452-5p upregulation promotes the tumorigenicity of BC cells, thus revealing that miR-452-5p facilitates the progression of BC.

The lncRNA-miRNA-mRNA network has been extensively studied as biomarkers and potential therapeutic targets for BC diagnosis and prognosis [25, 38]. For instance, BCRT1 promotes BC progression by sponging miR-1303 and upregulating PTBP3 [39]. OIP5-AS1 and miR-216a-5p/GLO1 form a competitive endogenous RNA (ceRNA) regulatory network in BC, thus promoting the malignant behavior of cancer cells [40]. In my current work, I further explored miR-452-5p's interaction with LINC01140. Targeting analyses uncovered the miR-452-5p's binding to LINC01140. More interestingly, I learned that miR-452-5p levels were negatively associated to that of LINC01140 in BC tissues. This further supports that LINC01140 has a sponging effect on miR-452-5p. Salvage analyses exhibited that overexpressing LINC01140 counterbalanced the malignant migration and proliferation of BC cell lines after the increase of endogenous miR-452-5p. Therefore, I hypothesized that LINC01140 could inactivate miR-452-5p by sponging it to inhibit the induction of malignant BC cell behavior.

The abnormal regulation of RGS2 has been associated with the development of solid tumors. Also, the downregulation of RGS2 has been reported in the progression of various cancers [23, 24, 41]. RGS2 is underexpressed in BC; its overexpression can inhibit epidermal growth factors or the serum-induced proliferation of cancer cells [24]. BC patients with poor levels of RGS2 had notably lower overall survival as RGS2 operates as a suppressor gene in BC [42]. In my current work, I confirmed the low expression of RGS2 in BC. I also revealed that RGS2 overexpression inhibited the malignant behavior of BC cells, and that it had an anticancer effect in BC. Moreover, I evidenced that RGS2 was miR-452-5p's target gene and was involved in the proliferation and migration of BC cells, which were regulable via the LINC01140/miR-452-5p axis.

Given the limitations of my study, it is clear that further research needs to be done. Cumulative studies have documented abnormal miRNA cluster expressions in BC, showing protumor and antitumor effects [32]. In future studies, I may explore the effects of miR-452 clusters on BC, based on the report of Li et al. [35]. Furthermore, the downstream signaling pathway of the LINC01140/miR-452-5p/RGS2 axis is also worth investigating.

In conclusion, I emphasize the important role of LINC01140 in the emergence and progression of BC. My study is the first to demonstrate that LINC01140 is involved in BC migration and survival through the miR-452-5p/RGS2 axis. The findings from this study contribute new insights on the mechanisms of BC progression and suggest potential targets for its treatment.

## Figures and Tables

**Table 1:** Primers used in this study.

Name	Primer	Sequence(5-3′)
LINC01140	Forward primer	CATCTCATCGGCATGGACCT
	Reverse primer	CAAACTGGACTGACTTTCACCA
miR-452-5p	Forward primer	AGCGCGAACTGTTTGCAGAGGA
	Reverse primer	ATCCAGTGCAGGGTCCGAGG
RGS2	Forward primer	AGTAAATATGGGCTGGCTGCATTC
	Reverse primer	GCCTCTTGGATATTTTGGGCAATC
GAPDH	Forward primer	GGAGCGAGATCCCTCCAAAAT
	Reverse primer	GGCTGTTGTCATACTTCTCATGG
U6	Forward primer	CTCGCTTCGGCAGCACA
	Reverse primer	AACGCTTCACGAATTTGCGT

## Data Availability

The datasets that have been used and/or analyzed during the current study are available from the corresponding author upon reasonable request.

## Supplementary Materials

Supplementary Materials Supplementary Table 1. The miRNAs binding to LINC01140 predicted by starBase.
