# Supplementary material for: LINC01140 Targeting miR-452-5p/RGS2 Pathway to Attenuate Breast Cancer Tumorigenesis
Source: Dis Markers. 2022 Oct 17;2022:2434938. doi: 10.1155/2022/2434938 (PMC9592237; doi:10.1155/2022/2434938)
Supplement: Supplementary Materials — Supplementary Table 1. The miRNAs binding to LINC01140 predicted by starBase. [file 2434938.f1.docx]

Supplementary table 1. The miRNAs binding to LINC01140 predicted by starBase.

| miRNA name | Gene Name | chromosome | start | end |
| --- | --- | --- | --- | --- |
| hsa-miR-4676-3p | LINC01140 | chr1 | 87600063 | 87600086 |
| hsa-miR-892c-3p | LINC01140 | chr1 | 87600065 | 87600086 |
| hsa-miR-452-5p | LINC01140 | chr1 | 87600066 | 87600086 |
| hsa-miR-200c-3p | LINC01140 | chr1 | 87600067 | 87600089 |
| hsa-miR-200b-3p | LINC01140 | chr1 | 87600068 | 87600089 |
| hsa-miR-429 | LINC01140 | chr1 | 87600068 | 87600089 |
| hsa-miR-122-5p | LINC01140 | chr1 | 87600130 | 87600147 |
| hsa-miR-376c-3p | LINC01140 | chr1 | 87600135 | 87600155 |
| hsa-miR-3140-3p | LINC01140 | chr1 | 87631620 | 87631641 |
